# The Resistance Patterns in *E. coli* Isolates among Apparently Healthy Adults and Local Drivers of Antimicrobial Resistance: A Mixed-Methods Study in a Suburban Area of Nepal

**DOI:** 10.3390/tropicalmed7070133

**Published:** 2022-07-12

**Authors:** Abha Shrestha, Rajeev Shrestha, Pramesh Koju, Sudichhya Tamrakar, Anisha Rai, Priyanka Shrestha, Surendra Kumar Madhup, Nishan Katuwal, Archana Shrestha, Akina Shrestha, Sunaina Shrestha, Sandip K.C, Supriya Kharel, Pooja Tamang, Pruthu Thekkur, Sony Shakya Shrestha

**Affiliations:** 1 Department of Community Medicine, Kathmandu University School of Medical Sciences, Kathmandu University, Dhulikhel 45210, Nepal; shresthabha@kusms.edu.np; 2 Department of Pharmacology, Kathmandu University School of Medical Sciences, Kathmandu University, Dhulikhel 45210, Nepal; rajeev.shrestha@kusms.edu.np; 3 Pharmacovigilance Unit, Dhulikhel Hospital, Dhulikhel 45210, Nepal; kojupramesh@kusms.edu.np; 4 Research and Development Division, Dhulikhel Hospital, Dhulikhel 45210, Nepal; sudichhya@dhulikhelhospital.org (S.T.); anisharai@kusms.edu.np (A.R.); nishankatuwal@dhulikhelhospital.org (N.K.); supriya@kusms.edu.np (S.K.); poojatamang@dhulikhelhospital.org (P.T.); 5 Department of Public Health and Community Programs, Dhulikhel Hospital, Dhulikhel 45210, Nepal; archana@kusms.edu.np (A.S.); akinakoju@kusms.edu.np (A.S.); 6 World Health Emergencies Programme, WHO Country Office, Kathmandu 41825, Nepal; pshrestha@who.int; 7 Department of Microbiology, Dhulikhel Hospital, Dhulikhel 45210, Nepal; madhup@kusms.edu.np (S.K.M.); sunaina@dhulikhelhospital.org (S.S.); 8 Health Unit, Dhulikhel Municipality, Dhulikhel 45210, Nepal; info@dhulikhelmun.gov.np; 9 Centre for Operational Research, International Union Against Tuberculosis and Lung Disease (The Union), 75000 Paris, France; pruthu.tk@theunion.org

**Keywords:** antimicrobial resistance, local drivers, mixed-method study, multi-drug resistance

## Abstract

Evidence-based decision-making to combat antimicrobial resistance (AMR) mandates a well-built community-based surveillance system for assessing resistance patterns among commensals and pathogenic organisms. As there is no such surveillance system in Nepal, we attempted to describe the antimicrobial resistance pattern in *E. coli* isolated from the fecal samples of apparently healthy individuals in Dhulikhel municipality and also explored the local drivers of AMR. We used a mixed-method design with a cross-sectional quantitative component and a descriptive qualitative component, with focus group discussion and key informant interviews as the data collection method. Fecal samples were collected from 424 individuals randomly selected for the study. *E. coli* was isolated from 85.9% of human fecal samples, of which 14% were resistant to ≥3 class of antimicrobials (multidrug resistant). Of the 368 isolates, resistance to ampicillin (40.0%), tetracycline (20.7%) and cefotaxime (15.5%) were most prevalent. The major drivers of AMR were: lack of awareness of AMR, weak regulations on sales of antimicrobials, poor adherence to prescribed medications, and incomplete dosage due to financial constraints. These findings indicate the need for strict implementation of a national drug act to limit the over-the-counter sales of antimicrobials. Additionally, awareness campaigns with a multimedia mix are essential for educating people on AMR.

## 1. Introduction

Antimicrobial resistance (AMR) is one of the leading causes of death (estimated five million associated deaths annually) globally [1]. In recent years, AMR has made therapeutic drugs ineffective against bacterial infections [2]. The increasing levels of AMR are accompanied by limited reserves of antimicrobial drugs to tackle them. This endangers the sustainability of effective public health responses to infectious diseases with resistant organisms [3,4]. Increased patient morbidity, mortality, health-care-related costs, and treatment failure are key repercussions of this situation [5,6]. Furthermore, increased mortality risk due to a lack of effective antimicrobials could make procedures such as surgery, transplantation, and chemotherapy untenable [4,7].

*E. coli* is one of the most abundant commensals present in the intestine and is excreted in the feces of humans and animals [8]. It is an opportunistic pathogen that causes urinary tract, bloodstream, and food-borne infections, as well as meningitis in newborns [9]. Studies on the recent global burden of bacterial resistance reported that *E. coli* is the leading cause of death due to resistance. Multidrug resistance (MDR) intestinal commensals among healthy humans have been documented for several years, and the proportion with resistance has been steadily increasing in recent years [10]. The previous studies largely from hospital settings have reported high rates of MDR among *E. coli* isolates ranging from 29% to 45% [11,12,13,14]. *E. coli* being one of the major causes of infections globally, MDR could lead to many health-related complications and an economic burden [15].

The AMR in commensals could contribute to an increase in AMR among organisms pathogenic to humans [16]. In vivo, the resistance genes from commensals could be transmitted to pathogenic *E. coli* or Salmonella strains and vice versa [17]. Later, the cross-transmission of drug-resistant pathogens can infect other people and lead to community outbreaks. Similarly, hospitalized patients colonizing MDR commensal bacteria can lead to nosocomial infections [18,19]. Knowing the resistant patterns and burden of MDR among bacteria present in one’s gut could help predict the resistance profile of a subsequent clinical illness [20]. Thus, AMR surveillance programs have highlighted the importance of assessing resistance patterns in the commensal intestinal *E. coli* [21]. As resistance genes are prevalent in the fecal *E. coli* strains from healthy individuals, surveillance of resistance in the community setting is necessary to forecast AMR trends [22].

Nepal, a landlocked country in South Asia, has a high burden of AMR. The density of medical doctors and pharmacists per 10,000 population is lower in Nepal than in the neighboring countries like India, Bangladesh, and Pakistan [23]. The irrational use of antimicrobials, the scarcity of bacterial confirmation and susceptibility testing, the lack of well-equipped facilities, and a poor physician-to-patient ratio all contribute to the high burden of AMR [24]. The National Action Plan for Containment of Antimicrobial Resistance 2016 envisions rigorous and nationwide surveillance for AMR [25]. The AMR surveillance program started in 1999. Since 2004, the National Public Health Laboratory has been coordinating the AMR surveillance with technical support from WHO [26]. However, surveillance is limited to only a few bacterial pathogens from 21 laboratories [27]. Thus, there is no information on the antibiogram of intestinal commensals in Nepal.

As most of the studies conducted in Nepal concentrate on the resistance patterns of microorganisms in the hospital setting, to the best of our knowledge, no studies have tried to understand the local drivers of AMR in the community. Understanding how AMR is associated with various drivers along with the resistance patterns is critical for strategic planning in the community. In this regard, we aimed to identify patterns of drug resistance in the *E. coli* present in apparently healthy adult fecal samples in selected wards of Dhulikhel municipality and the possible local drivers of AMR in humans.

## 2. Materials and Methods

### 2.1. Study Design

We adopted a concurrent mixed-methods design. The quantitative component was a cross-sectional study involving primary data collection among apparently healthy individuals to assess the antimicrobial resistance pattern of *E. coli* in fecal samples. The qualitative component was a descriptive study with focus group discussions (FGDs) and key informant interviews (KIIs) as a data collection method.

### 2.2. Study Setting

The study was conducted in Dhulikhel municipality in the Kavrepalanchowk district of central Nepal. Nepal is a Federal Democratic Republic country in Asia, mainly situated in the Himalayas but also including parts of the Indo Gangetic plain [28]. According to the 2021 census, Nepal has a population of 29,192,480 [29], with the majority of people involved in agriculture as their primary occupation. The National Health Policy of Nepal (2014) aims to improve access to quality and equitable health services, provide basic health services free of cost, and cover other services through social health insurance [30]. Although the AMR surveillance program started in 1999, as in many countries, the system is suboptimal in Nepal, resulting in under-reporting [31].

Dhulikhel municipality is 30 km southeast of Kathmandu and has a population of 32,026. The total literacy rate is 75.26%. The municipality is administratively divided into 12 wards (clusters). It has one tertiary-level hospital (Dhulikhel Hospital-Kathmandu University Hospital), one primary health center, six health posts, and three urban health posts. So far, there are only a few animal clinics within Dhulikhel municipality.

### 2.3. Study Population and Sampling

Quantitative:

All individuals above 18 years of age living in Dhulikhel municipality for at least six months who consented to participate were eligible for the study. The sample size was calculated using $Z_{\left(1-\alpha /2\right)}$ as a standard normal variate (1.96 at 5% type I error (*p* < 0.05)), where absolute precision or error (d) was taken as 5% at type I error of 5%. Since the prevalence of *E.coli* was unknown among apparently healthy individuals, it was considered to be 50% [32]. The minimum of 424 apparently healthy individuals was supposed to be approached for the study after accommodating a 10% non-response rate. The sample size was calculated using the formula below:

(1) $$Sample\;size=\frac{Z_{\left(1-\alpha /2\right)}{}^{2}p\left(1-p\right)}{d^{2}}$$

where $Z_{\left(1-\alpha /2\right)}$ is a standard normal variate, p is expected proportion in population, and d is absolute error or precision.

We conveniently chose two of the twelve wards (one rural and one semi-urban) that were closer to Dhulikhel Hospital. The number of individuals recruited from each ward was proportional to the total number of households in each ward. We used systematic random sampling to select the houses in each ward. We selected the first household using the spin-the-bottle method and followed the right-hand rule to select every second household until the sample size was met. Then, we selected one member from the household using the Kish technique. Those under medication, those who did not give written consent, and those who frequently moved in and out of the municipality were not included in the study.

Qualitative:

To identify local drivers of AMR, we included a diverse group of participants to reflect different points of view. A list of pharmacies, food vendors, health coordinators, and community members was obtained from Dhulikhel municipality as these groups were involved in the phenomenon of interest of this study; these groups represented the health sector, community, and policy makers, all of whom have significant roles in determining AMR. We selected the participants using purposive sampling based on the inclusion criteria: age above 18 years, currently living in Dhulikhel, and willing to participate in the study. To develop a meaningful concept of the local drivers, we conducted three FGDs (with 12 participants in each group) and 22 KIIs.

### 2.4. Study Variables, Sources and Data Collection

Quantitative:

We interviewed the study participants using a structured questionnaire to collect demographic details such as age, sex, geographical location, hospitalization status, travel history, and previous antimicrobial use. Fecal samples were collected and transported to the microbiology laboratory in a biohazard box. Each sample was given a unique identification number. Samples were inoculated in MacConkey agar plates and incubated at 37 °C for 24 h. Lactose-fermenting colonies were selected and subcultured in MacConkey agar. Gram-staining and biochemical tests (oxidase test, catalase test, citrate utilization test, urease test, sulfur indole motility (SIM) test, triple sugar iron (TSI) test) were performed for the confirmation of *E. coli*. Different isolates of *E. coli* were identified based on their colony morphology, motility and AST pattern. For antimicrobial sensitivity testing, the Kirby–Bauer disc diffusion method was performed, which included the preparation of a microbial suspension of *E. coli* followed by the preparation of 0.5 McFarland standard. The suspension was cultured onto Muller–Hinton agar to obtain a confluent lawn. The antibiotic discs were then placed on the agar surface. The plates were observed for the zone of inhibition (mm) after incubation for 16–18 h [33]. The decision on sensitivity and resistivity was made per the manufacturer’s instruction for each antibiotic tested. All procedures followed Clinical and Laboratory Standard Institute (CLSI) guidelines [34].

The *E. coli* isolates were tested against cefotaxime, ciprofloxacin, ampicillin, tetracycline, chloramphenicol, gentamicin, and cotrimoxazole. Isolates showing resistance to more than two classes of antimicrobials were considered MDR isolates.

Qualitative:

We conducted FGDs and KIIs to collect qualitative data to identify the local drivers of AMR. The face-to-face interviews provided an opportunity to probe specific issues noted during the FGD [35,36]. In this study, we organized a workshop initially to build the capacity of research assistants to conduct the interviews. Before the interview, we contacted all the participants through telephone calls following a standard script to obtain verbal consent. Participation in the study was voluntary, and there were no financial incentives. Informed written consent was obtained from the participants. We developed separate guides to conduct the KIIs and FGDs among different study groups. These guides were pretested before being used among the study participants. The trained researchers conducted the interviews and the discussions in the Nepali language. The interviews lasted about 35 min, and the FGDs lasted between 60 and 90 min. With the consent of the participants, we recorded the audio and took notes on the discussion. The participants had the right to withdraw anytime during the interview. The information regarding participants has been kept confidential and anonymized.

### 2.5. Data Analysis

The collected data were entered in Epidata software v3.1 (EpiData Association Odense, Denmark). Data entry, cleaning, and coding were supervised by the research team and cross-validated by the principal investigator. Data were analyzed using Stata software v12.1 (Stata Corp, College Station, TX, USA). Laboratory results along with demographic data were entered in a Microsoft Excel spreadsheet, and resistance patterns of *E. coli* were shown through frequencies and proportions. The isolates with resistance to three or more classes of antimicrobials were considered MDR bacteria. Univariate log-binomial regression was used to assess the individual-level factors associated with the presence of *E. coli* and MDR. Prevalence ratios (PR) with 95% confidence interval (CI) were used as a measure of association. The level of significance was set at *p* ≤ 0.05.

We gave unique codes to participants from the FGDs and KIIs to maintain confidentiality. We transcribed and translated the recorded audio from the FGDs and KIIs into English. We developed a code book based on the questions asked during the interviews and group discussions. Manual content analysis was conducted to deduce codes, and the codes were entered in Excel. Thematic analysis was performed using the codes, and verbatim quotes relevant to the codes are presented. Several codes related to the local drivers of AMR were further grouped into categories.

## 3. Results

### 3.1. Quantitative Findings

**Figure 1:**
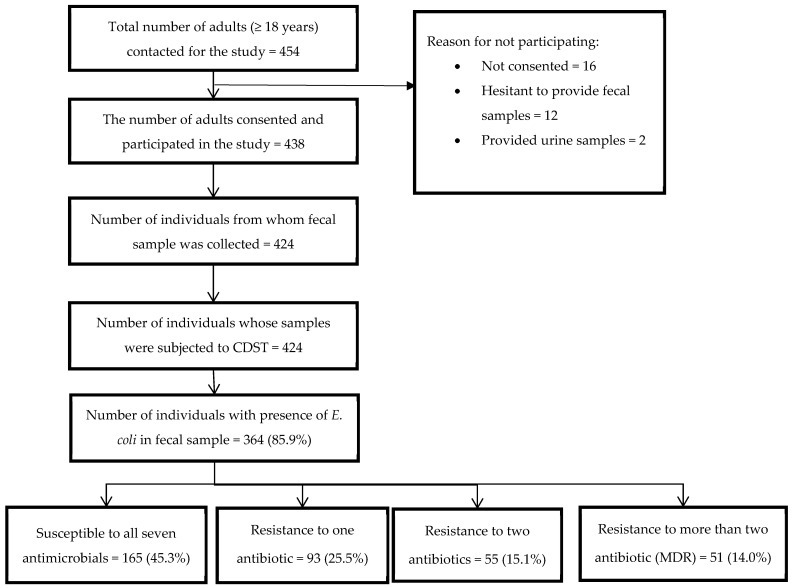
Flow chart depicting the patterns of resistance in *E. coli* in fecal samples from healthy humans and resistance to antimicrobials among adults in Dhulikhel municipality in Nepal from September to December 2021.

A total of 454 adults were approached for participation in the study. Out of the 454 contacted, 424 (96.8%) provided a fecal sample. *E. coli* was isolated from the fecal samples of 364 (85.9%) individuals. Among those isolates, 165 (45.3%) isolates did not exhibit resistance to any of the seven antimicrobials used in the study, while 51 (14.0%) isolates were MDR (resistant to ≥3 class of antimicrobials) (Figure 1).

The mean age (SD) of the participants was 46 (14.4) years, and 291 (68.6%) were female. More than half (56.1%) of the participants had no formal education, and the majority (75.5%) relied on agriculture or livestock for their livelihood. Of the total participants, 67% lived in houses with water piped into their yard, and <1% did not have proper toilet facilities. Almost all the participants said they adhered to hand-washing practices before cooking and eating and after toilet use. Only 3 (0.7%) individuals claimed to consume water from open sources (Table 1).

On univariate analysis, individuals from Janajati (1.1, 95% CI: 1.0–1.2) compared with Brahmin and those from the Buddhist religion (1.1, 95% CI: 1.0–1.2) had higher prevalence of *E. coli*. Similarly, individuals with no access to improved sanitation (1.2, 95% CI: 1.1–1.2) and who consumed water from open sources (1.1, 95% CI: 1.0–1.2) had higher prevalence of *E. coli* (Table 1).

In total, 368 isolates of *E. coli* were found among 364 individuals, with 2 individuals having 2 isolates in the sample provided. Of the 368 isolates, resistance to ampicillin (40.0%), tetracycline (20.7%), and cefotaxime (15.5%) were most prevalent. Only 2 (0.5%) isolates out of 368 were resistant to gentamicin (Table 2).

Out of *E. coli* isolated from the 424 collected fecal samples, 51 (14.0%) were reported to be resistant to three or more than three antimicrobials used in this study, although on univariate analysis, none of the sociodemographic or environmental factors were associated with having MDR (Table 3).

### 3.2. Qualitative Findings

**Figure 2:**
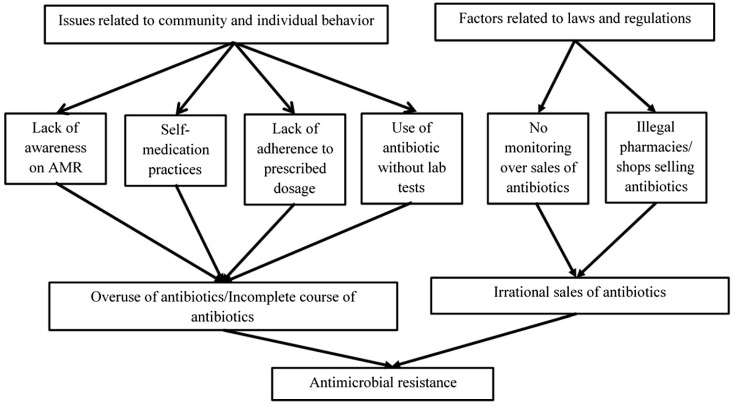
Local drivers of AMR as perceived by ward chairpersons, policy makers, pharmacists, community members, and health in-charges in Dhulikhel municipality, 2021.

The findings from the FGDs and KIIs were grouped into major categories: Issues related to community and individual behavior and factors related to laws and regulations (Figure 2).

#### 3.2.1. Issues Related to Community and Individual Behavior

In this study, the interviewees highlighted four major issues related to community and individual behaviors as the potential drivers of AMR in humans. The challenges identified are described below:

1. Lack of awareness on antimicrobials and AMR among community members

Though the health care workers, ward chairpersons, and policy makers had an idea about AMR, it was found that most of community members were unaware of it. Most of the community members perceived antimicrobials as medicines that would cure disease faster or as medicine that is used when other medications fail to work. Most local people believed that a full course of antimicrobials should be completed, although on the contrary, one individual felt that antimicrobials could be discontinued once they started feeling better. Even though most community members thought that a full course of antimicrobials should be consumed, none had ever heard of AMR or its causes or consequences:

*Yes, the doctor prescribes antimicrobials. But I think we can discontinue the medicines once the symptoms subside. I don’t feel like continuing the medicine once I start feeling better. P(3)*

*The main problem is that the patient is not aware that the full dose of the medicine needs to be completed, when symptoms subside after having 2-3 tablets, they throw or stop taking the remaining medicine. They are not aware of the consequences. I feel public awareness is the most important thing needed. (WC_6)*

2. Self-medication practices

The participants reported the presence of self-medication practices in the community. Old prescriptions and leftover medicines were believed to be shared among family members with similar symptoms without any medical consultation. People apparently buy the medicines from pharmacies using old prescriptions and consume them:

*What people usually do is, if they have symptoms similar to previously cured illness, they visit the drugstore directly and ask for the same old medicine, which increases the frequency of using the medicine even though it is not required. (FGD2-P13)*

The pharmacists explaining the issue said that they receive requests for antimicrobials without a prescription even from some health care workers:

*We ask if the doctor has written any prescription or have they been taking the antimicrobials regularly. Most of them practice self-medication and some of them are health care workers too. (P001)*

3. Financial constraints

The participants revealed that poor financial status had impacts on the use of antimicrobials. The interviewees perceived that the cost of consultation, various laboratory tests, and antimicrobials might not be affordable and considered that individuals preferred buying antimicrobials directly from shops/pharmacies rather than going for a consultation to avoid the economic burden. As patients cannot afford doctor visits for the prescriptions for appropriate antimicrobials, these are hardly performed, leading to the empirical prescription of antimicrobials:

*A culture test determines whether the antimicrobial will work for the particular disease or not, but it is very expensive. A general culture will cost you more than 1200 and the report will be ready only after 2-3 days. In such a case, it becomes difficult for an individual to afford the cost for laboratory investigations. (H001)*

The interviews and discussions highlighted financial constraints as one of the reasons for poor compliance with prescribed antimicrobials. Individuals fail to purchase an entire course of prescribed antimicrobials due to high out-of-pocket costs. In such situations, there will be a lack of adherence to the prescribed medication. A pharmacist admitted to the same and added,

*Some of these antimicrobials are a little expensive, they can’t afford a complete course of medicine, and they can’t take it all at once. One course can cost him up to Rs1500-2,000. He may not have the money to complete this course, so he takes one half course of antimicrobials. (P002)*

4. Overuse of antimicrobials/Incomplete course of antimicrobials

As manifested in the interviews and discussions, financial constraints significantly impact completing courses of antimicrobials. It was also reported that people approach pharmacies rather than going for a consultation to avoid the expenditure. The community members were unaware of antimicrobial resistance, leading to the unnecessary use of antimicrobials:

*People visit medical/pharmacy even if they have a simple fever and the pharmacist gives antimicrobials to them without proper check-ups and urine/fecal examinations. They are overusing antimicrobials. They think if they visit the hospital instead, it will cost a lot for all examinations. That’s why antimicrobials are being overused. I guess there is no single person in the village who has not taken antimicrobials. (WC_1)*

#### 3.2.2. Factors Related to Laws and Regulations

Nearly all the participants perceived that laws and regulations are required to prevent irrational sales of antimicrobials. On the contrary, the health in-charges stated that there were no laws to prevent the inappropriate selling of antimicrobials. It was also revealed from the FGDs and KIIs that illegal shops/pharmacies dispense medicines, including antimicrobials. Under this category, two subcategories have been identified:

1. Lack of guidelines to monitor the selling of antimicrobials

The participants in the KIIs complained that antimicrobials are being sold without prescription in the absence of laws and guidelines. Antimicrobials are also prescribed even for simple diseases without proper laboratory investigations. During one of the interviews, a pharmacist mentioned that there is no guideline for selling antimicrobials and they sell it based on their experience. A policy maker expressed a similar opinion regarding the lack of specific guidelines for effective monitoring. According to the participants from KIIs, this is the reason for the irrational sales and use of antimicrobials:

*The medicine shops recommend them to take some strong medicine that is why people here use lots of antimicrobials......There is a system prevalent that even for a simple type of disease, people go to medicinal shops in Banepa and then take strong antimicrobials..........there is no monitoring or surveillance because they take medicine from medicine shops without tests and prescriptions, there is such a system in Nepal that is why people openly use antimicrobials. (H002)*

2. Unregistered pharmacies/shops

The participants in the KIIs showed concern about the selling of antimicrobials by unregistered shops and unlicensed persons. Some participants stated that antimicrobials are being sold even at grocery stores and pesticide shops, especially in rural areas. Such illegal activities encourage the unnecessary use of antimicrobials:

*In terms of resistance, unregistered pharmacies should be taken under control….I have seen grocery stores in Dhulikhel selling paracetamols, syrups and amoxicillin. I was surprised to see that. Such practices should not be allowed at any cost. (H003)*

*Yes. It is not ‘might be selling’, such unlicensed persons are selling antimicrobials and other drugs. We have found it once or twice. (WC-6)*

3. Irrational sales and use of antimicrobials

According to the participants in the KIIs, one of the major challenges related to AMR is the irrational sale and use of antimicrobials. This results in the prescription of unnecessary dosages of antimicrobials and overuse of antimicrobials on the consumer side:

*If we go to the pharmacy for the common cold, they will recommend a lot of antimicrobials. It is said that patients with cold do not have to take any medicine, as they will recover in seven or ten days without medicine. Nowadays, the pharmacy will directly prescribe you strong medicine like azithromycin. When using such drugs, we should think twice. They lie that we sell drugs on prescription if we go for inspection. This is challenging because the truth is hidden. And another challenge is that consumers these days are very clever because people themselves come to buy Amoxicillin and ranitidine directly without a prescription. Grocery stores also sell antimicrobials. Because of these things we are facing a lot of challenges. (PM002)*

## 4. Discussion

This first community-based study from Nepal on the resistance patterns of *E. coli* among apparently healthy individuals showed that more than two thirds of the participants had *E. coli* in their fecal samples. Among the *E. coli* isolated, one in four isolates was resistant to at least one of the seven antimicrobials tested, and one in eight had MDR (≥3 class of antimicrobials). The highest resistance was observed for ampicillin and tetracycline, whereas the lowest resistance was found against gentamicin. Lack of awareness of AMR, lack of regulatory measures to monitor the sale of antimicrobials, and prevalent self-medication practices in the community were perceived to be the local drivers of AMR.

The estimation of five million annual global AMR deaths highlights the importance of AMR control through decisions based on routine surveillance and local drivers of AMR [1]. Surveillance for data-driven decisions to control AMR is relevant to meeting the Sustainable Development Goals (targets 3.1 to 3.3 and 3.8 and those of Goal 6), especially that focused on reducing AMR (3.d.2) [37]. This study supplements the global call for assessing the patterns of AMR in *E. coli* at the community level, specifically in Nepal. In the future, these study findings can be used as baseline information to compare the resistance patterns detected through the routine community-level AMR surveillance.

This study sheds light on the relatively high rate of MDR in the *E. coli* isolated from healthy human feces, which showed high resistance to commonly used drugs and antimicrobials. The study also indicates that lack of awareness, overuse/misuse of antimicrobials, and irrational sales of antimicrobials are the leading causes of AMR in this community. These warrant swift regulation of antimicrobial prescribing and dispensing practices by applying strict legislation to avoid further progression of AMR. In addition, educating the community on appropriate antimicrobial use and consequences of AMR is also essential.

The study has some strengths. As we adopted a community-based mixed-method design, it allowed for the estimation of the burden of resistance and an understanding of the local drivers of AMR. There was less scope for selection bias as we used systematic random sampling and the recruitment rate was high (96.5%). Finally, we adhered to the COREQ and STROBE checklists for reporting qualitative and quantitative findings, respectively. However, conducting a study in only one municipality of the country (lack of generalizability) and the inability to classify the *E. coli* isolates as commensal or pathogenic were the major limitations of the study.

The study findings have some programmatic implications. First, the current study shows the more than two thirds of the healthy individuals had *E. coli*. Similar to this study, a high prevalence of *E. coli* in human fecal samples has been reported in Nigeria (88%), northern California (90.3%), southern Taiwan (66.3%), Bangladesh (82%), and Congo (51%) [8,38,39,40]. However, future studies must look at the proportions of pathogenic *E. coli* among apparently healthy individuals as these can cause outbreaks.

Second, the initiation of a surveillance system to monitor the burden of *E. coli* and its pathogenic strains would help to understand the local transmission of the pathogens [41]. Establishing such a surveillance system seems feasible as >95% of the apparently healthy individuals were willing to participate in the study and provided a fecal sample. Furthermore, commensal *E. coli* bacteria may constitute a potential reservoir of AMR genes that can be transferred to other bacterial species [42,43].

Third, similar to our findings, studies in the past have reported high resistance to ampicillin (60–90%) and tetracycline (74%) in *E. coli* isolates [36,40,44,45]. Even the community-based study among apparently healthy individuals in India reported that 75% of the *E. coli* isolates were resistant to ampicillin [46,47]. Furthermore, almost one sixth of the *E. coli* isolates were resistant to cefotaxime, a third-generation cephalosporin that is widely used in the empirical treatment of patients with suspected Gram-negative community-acquired bacteria [48]. Compared with previous studies, the percentage of resistance to third-generation cephalosporin was higher in the current study [49,50,51,52]. *E. coli* resistant to third-generation cephalosporin causes the highest mortality globally among all the resistant bacteria combinations and is associated with extended hospitalization and higher expenses [53].

Fourth, as found in the current study, community-based studies from Africa (2%), Congo (0%), South Africa (2%), and Nigeria (7.7%) reported low resistance to gentamicin [36,44,54,55]. Gentamicin is primarily administered by parenteral routes, including systemic, topical, and ophthalmic formulations, owing to its minimal gastrointestinal absorption. As a result, it is rarely employed in a community setting [56]. On the contrary, a study conducted among the patients of an intensive care unit with suspected health care associated infection reported that 64.5% of the *E. coli* isolates were resistant to gentamicin [57]. This may be due to the high chance of acquiring resistant genes and their ability to persist and multiply in a hospital environment.

Fifth, the previous studies from southern Taiwan (37%), northern California (52%), and India (33–49%) reported higher percentages of MDR isolates than in our study [16,40,44,54,58]. This higher proportion of MDR might be due to the fact that the previous studies were hospital-based and had admitted patients as participants, whereas the current study included apparently healthy individuals from the community. The former has a higher chance of being exposed to the antimicrobials and higher potential to develop resistance. Disparities in resistance rates between studies could be attributable to differences in antimicrobial prescribing for different age groups, seasonal changes, prescription duration, and even patient compliance with prescribed antimicrobials [59]. The studies on patterns of AMR have reported that high levels of resistance are seen against those antimicrobials that are more affordable and easily accessible [44]. Although prior antimicrobial use may contribute to variances in antimicrobial resistance, other factors like the characteristics of the patients as well as socioeconomic and geological factors may also influence the variations in MDR [60]. However, in this study, the proportion of MDR did not significantly vary across sociodemographic or environmental factors; none of the factors was associated with the MDR.

Sixth, the qualitative exploration revealed widespread irrational use of antimicrobials that was perceived as one of the major reasons for AMR in the municipality area. The perceived irrational use of antimicrobials was similar to that reported in other low- and middle-income countries [46,61,62,63,64,65,66]. The study participants’ perception could be close to the truth, as thirteen studies on antimicrobial prescribing patterns in Nepal reported that patients were prescribed antimicrobials without any bacterial confirmation or susceptibility testing [25]. Though the National Drugs Act 2038 of Nepal categorized antimicrobials in category B, drugs that should only be sold by prescription and by qualified personnel, its enforcement is deficient [67]. Therefore, there is an urgent need to regulate antimicrobial prescription and dispensing in line with Antimicrobials Treatment Guidelines 2014 by strictly enforcing the National Drugs Act 2038 and implementing nationwide surveillance of antimicrobial use [25].

Seventh, the health care workers and the general public felt that AMR is mainly due to poor awareness about AMR and also irrational use of antimicrobials. Similar to our findings, studies have reported that although people were familiar with antimicrobial drugs, the majority were unaware of AMR and the importance of adhering to prescribed antimicrobials. Thus, individuals fail to adhere to the prescribed dose or duration of the antimicrobials [68,69,70]. However, as mentioned in the current study participants, poor economic status contributes to the irrational use of antimicrobials as patients avoid expensive medical consultation and laboratory investigations. Instead, they prefer buying antimicrobials directly from pharmacies or medical shops without a prescription. In Nepal, most people find it challenging to afford antimicrobials prescribed for a longer duration, thus leading to incomplete courses [71]. Instead, patients cease taking medications as soon as the disease symptoms fade away and typically save the remaining medications for future use, contributing to the development of the resistance [72,73,74].

Finally, the health care workers in this study highlighted the need to improve the surveillance system for AMR in Dhulikhel. A study conducted in Bagmati province assessed the quality of AMR data supplied to the National Public Health Laboratory (NPHL) of Nepal from five AMR surveillance sites. Unfortunately, despite considerable volume, the data lacked completeness and consistency [75]. Such deficient AMR surveillance systems fail to inform health care providers about the circulating resistant microorganisms and the effective treatment regimens to be used.

## 5. Conclusions

This study indicates that almost one sixth of isolates of *E. coli* from the fecal samples of a group of apparently healthy human individuals had multidrug resistance (≥3 class of antimicrobials). Resistance was high toward commonly used drugs such as ampicillin and tetracycline. Some of the major drivers of AMR were poor awareness about AMR, irrational sales of antimicrobials due to weak regulatory measures, and poor adherence to prescribed drugs due to financial constraints. These findings indicate the need for strict implementation of the national drug act to limit the over-the-counter sales of antimicrobials. Additionally, awareness campaigns with a multimedia mix for educating people on AMR are warranted.

## Figures and Tables

**Table 1:** Sociodemographic and environmental factors associated with presence of *E. coli* among adults of Dhulikhel municipality of Nepal from September to December 2021.

Characteristics	Total		*E. coli*		Unadjusted		*p* Value
	N	(%) ^	n	(%) ^$^	PR	(95% CI)	
Total	424	(100)	364	(85.9)			
Age (in years)							
18–29	56	(13.2)	48	(85.7)	1.0	(0.9–1.2)	0.611
30–44	151	(35.6)	131	(86.8)	1.1	(0.9–1.2)	0.400
45–59	131	(30.9)	114	(87.0)	1.1	(0.9–1.2)	0.380
≥60	86	(20.3)	71	(82.6)	1		
Gender							
Male	133	(31.4)	112	(84.2)	1		
Female	291	(68.6)	252	(86.6)	1.0	(0.9–1.1)	0.526
Marital status							
Never married	19	(4.5)	14	(73.7)	1		
Currently married/cohabitating	366	(86.3)	317	(86.6)	1.2	(0.9–1.5)	0.244
separated/divorced/widow	36	(8.5)	30	(83.3)	1.1	(0.8–1.5)	0.431
Refused to answer	3	(0.7)	3	(100.0)	1.4	(1.0–1.8)	0.026
Ethnicity/Caste							
Brahmin	175	(41.3)	143	(81.7)	1		
Chhetri	53	(12.5)	44	(83.0)	1.0	(0.9–1.2)	0.825
Janajati	176	(41.5)	159	(90.3)	1.1	(1.0–1.2)	0.021
Dalit	20	(4.7)	18	(90.0)	1.1	(0.9–1.3)	0.243
Religion							
Hindu	337	(79.5)	283	(84.0)	1		
Buddhist	73	(17.2)	68	(93.2)	1.1	(1.0–1.2)	0.009
Others ^#^	14	(3.3)	13	(92.9)	1.1	(0.9–1.3)	0.197
Education status							
No formal education	238	(56.1)	205	(86.1)	10	(0.9–1.2)	0.755
Primary school ^1^	75	(17.7)	65	(86.7)	1.0	(0.9–1.2)	0.724
Secondary school ^2^	58	(13.7)	49	(84.5)	1		
Higher secondary and above ^3^	53	(12.5)	45	(85.0)	1.0	(0.9–1.2)	0.951
Occupation status							
Agriculture/livestock	302	(75.5)	263	(87.1)	1.2	(0.9–1.7)	0.141
Business	45	(10.6)	39	(86.7)	1.2	(0.9–1.7)	0.176
Employed *	20	(4.7)	14	(70.0)	1		
Unemployed	57	(13.4)	48	(84.2)	1.2	(0.9–1.6)	0.240
Ward							
Ward-2	309	(72.9)	266	(86.9)	1.0	(0.9–1.1)	0.823
Ward-6	115	(36.6)	98	(85.2)	1		
Access to improved sanitation							
Yes	422	(99.5)	362	(85.8)	1		
No	2	(0.5)	2	(100.0)	1.2	(1.1–1.2)	<0.001
Presence of Livestock close to House							
Yes	329	(77.6)	287	(87.2)	1.1	(1.0–1.2)	0.173
No	95	(22.4)	77	(81.1)	1		
Consume water from open sources							
Yes	3	(0.7)	3	(100.0)	1.2	(1.1–1.2)	<0.001
No	421	(99.3)	361	(85.7)			

* Daily wage/labor, foreign employment, service (govt./private). ^#^ Kirat, Muslim, Christian. ^ Column percentage. ^$^ Row percentage. ^1^—till grade 5, ^2^—grade 5 to grade 10, ^3^—above grade 10.

**Table 2:** Patterns of resistance among the *E. coli* isolated from the fecal samples of adults in Dhulikhel municipality in Nepal from September to December 2021 (N = 368).

Antimicrobial	Sensitive		Indeterminate		Resistant	
	N	(%)	N	(%)	N	(%)
Cefotaxime	294	(79.9)	17	(4.6)	57	(15.5)
Ciprofloxacin	277	(75.3)	52	(14.1)	39	(10.6)
Tetracycline	289	(78.5)	3	(0.8)	76	(20.7)
Ampicillin	116	(31.5)	106	(28.8)	149	(40.5)
Chloramphenicol	319	(86.7)	36	(9.8)	13	(3.5)
Gentamicin	366	(99.5)	0	(0.0)	2	(0.5)
Cotrimoxazole	313	(85.1)	1	(0.3)	54	(14.7)

**Table 3:** Sociodemographic and environmental factors associated with multidrug resistance in *E. Coli* isolated from the fecal samples of adults in Dhulikhel municipality in Nepal from September to December 2021 (N = 364).

Characteristics	Total	MDR		Unadjusted		*p* Value
		N	(%) ^$^	PR	(95% CI)	
Total	364	51	(14.0)			
Age (in years)						
18–29	48	7	(14.6)	1.5	(0.6–4.0)	0.435
30–44	131	20	(15.3)	1.5	(0.7–3.5)	0.291
45–59	114	17	(14.9)	1.5	(0.7–3.5)	0.328
≥60	71	7	(9.9)	1		
Gender						
Male	112	17	(15.2)	1.1	(0.7–1.9)	0.668
Female	252	34	(13.5)	1		
Marital status						
Never married	14	1	(7.1)	1		
Currently married/Cohabitating	317	46	(14.5)	2.0	(0.3–13.7)	0.467
Separated/divorced/Widow	30	4	(13.3)	1.9	(0.2–15.2)	0.560
Refused to answer	3	0	(0.0)	-		
Ethnicity						
Brahmin	143	16	(11.2)	1		
Chhetri	44	7	(15.9)	1.4	(0.6–3.2)	0.402
Janajati	159	24	(15.1)	1.4	(0.7–2.4)	0.321
Dalit	18	4	(22.2)	2.0	(0.7–5.2)	0.170
Religion						
Hindu	283	42	(14.8)	1.3	(0.6–2.6)	0.521
Buddhist	68	8	(11.8)	1		
Others ^#^	13	1	(7.7)	0.7	(0.1–5.0)	0.676
Education status						
No formal education	205	29	(14.1)	1.4	(0.6–3.4)	0.476
Primary school ^1^	65	11	(16.9)	1.7	(0.6–4.5)	0.317
Secondary school ^2^	49	5	(10.2)	1		
Higher secondary and above ^3^	45	6	(13.3)	1.3	(0.4–4.0)	0.639
Occupation status						
Agriculture/livestock	263	34	(12.9)	1		
Business	39	6	(15.4)	1.2	(0.5–2.7)	0.670
Employed *	14	3	(21.4)	1.7	(0.6–4.7)	0.347
Unemployed	48	8	(16.7)	1.3	(0.6–2.6)	0.481
Ward						
Ward-2	266	33	(12.4)	1		
Ward-6	98	18	(18.4)	1.5	(0.9–2.5)	0.144
Access to sanitary latrine						
Yes	362	50	(13.8)	1		
No	2	1	(50.0)	3.6	(0.9–14.9)	0.074
Presence of livestock close to house						
Yes	287	36	(12.5)	1		
No	77	15	(19.5)	1.6	(0.9–2.7)	1.115
Consume water from open sources						
Yes	3	1	(33.3)	2.4	(0.5–12.2)	0.289
No	361	50	(13.8)	1		

* Daily wage/labor, foreign employment, service (govt./private). ^#^ Kirat, Muslim, Christian. ^$^ Row percentage. ^1^—till grade 5, ^2^—grade 5 to grade 10, ^3^—above grade 10.

## Data Availability

The data that support the findings of this study are available from the corresponding author upon a reasonable request.
